# Nanoencapsulation of general anaesthetics

**DOI:** 10.1039/d3na01012k

**Published:** 2024-02-15

**Authors:** Basma M. T. Abdoullateef, Saif El-Din Al-Mofty, Hassan M. E. Azzazy

**Affiliations:** a Department of Chemistry, School of Sciences and Engineering, The American University in Cairo New Cairo, AUC Avenue, SSE # 1184, P.O. Box 74 Cairo 11835 Egypt hazzazy@aucegypt.edu +20 226152559; b Department of Nanobiophotonics, Leibniz Institute of Photonic Technology Albert Einstein Str. 9 Jena 07745 Germany

## Abstract

General anaesthetics are routinely used to sedate patients during prolonged surgeries and administered *via* intravenous injection and/or inhalation. All anaesthetics have short half-lives, hence the need for their continuous administration. This causes several side effects such as pain, vomiting, nausea, bradycardia, and on rare occasions death post-administration. Several clinical trials studied the synergetic effect of a combination of anaesthetic drugs to reduce the drug load. Another solution is to encapsulate anaesthetics in nanoparticles to reduce their dose and side effects as well as achieve their sustained release manner. Different types of nanoparticles were developed as carriers of intravenous and intrathecal anaesthetics generating platforms which facilitate drug transport across the blood–brain barrier (BBB). Nanocarriers encapsulating common anaesthetic drugs such as propofol, etomidate, and ketamine were developed and characterized in terms of size, stability, onset and duration of loss of right reflex, and tolerance to pain in small animal models. The review discusses the types of nanocarriers used to reduce the side effects of the anaesthetic drugs while prolonging the sedation time. More rigorous studies are still required to evaluate the nanocarrier formulations regarding their ability to deliver anaesthetic drugs across the BBB, safety, and finally applicability in clinical settings.

## Abstract 2

General anaesthetics (etomidate, ketamine, propofol) may be encapsulated in different nanocarriers to facilitate their ability to cross the blood brain barrier while reducing their side effects.

## Graphical abstract

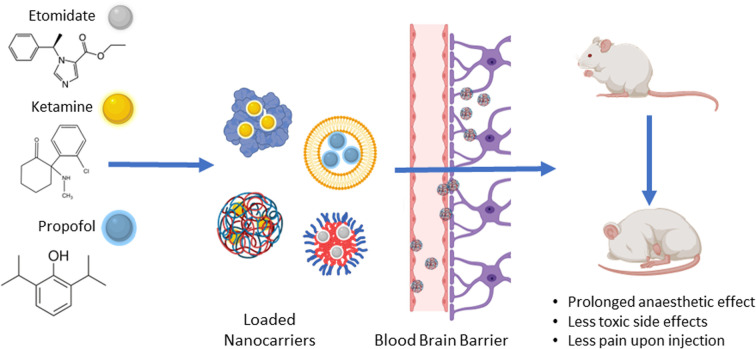

## Introduction

General anaesthesia, controlled loss of consciousness (LOC), is necessary to perform prolonged surgeries.^1^ General anaesthetic drugs are introduced intravenously to patients, at the beginning of surgery, to induce a rapid onset of sedation. Due to the short half-life of the drugs, sedation is maintained *via* further intravenous administration of anaesthetics or introduction of volatile anaesthetics (inhaled by the patient).^2^ Prolonged administration of anaesthetic drugs, however, causes several side effects, which may harm patients post-surgery, including hypotension, bradycardia, surgical infection, and thromboembolism.^3^ For selected surgeries, intrathecal or epidural anaesthesia were introduced to avoid unnecessary general anaesthesia.^4^ However, these routes aren't suitable for majority of surgeries such as open heart, tumour excision, and brain surgeries.

**Fig. 1:**
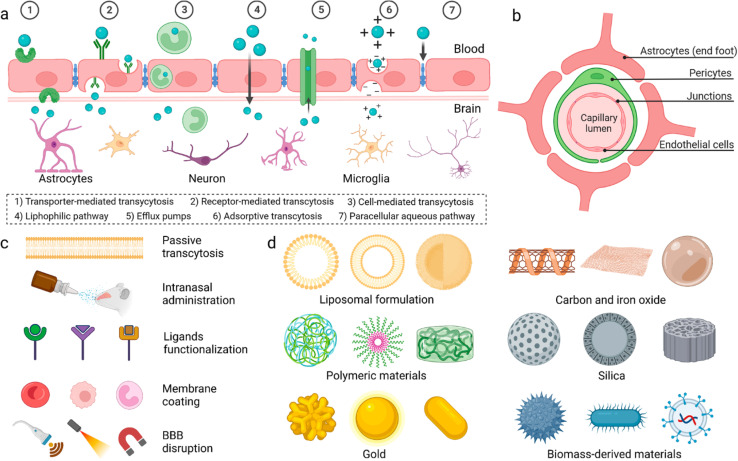
(a) Proposed mechanisms for crossing the blood–brain barrier (BBB). (b) Cellular composition of the BBB. (c) Methods utilized to enable materials to pass the BBB. (d) Different nanoparticles reported to have the ability to pass the BBB. Reproduced from Wu *et al.* with permission from [Springer-Nature], Copyright [2023].^7^

The blood–brain barrier (BBB) is made up of endothelial cells (bound together *via* tight junctions), pericytes, and astrocytes (Fig. 1). It regulates entry of metabolites and nutrients but prevents entry of harmful substances into the brain in order to maintain a suitable environment for proper function of neurons.^5^ Small (<500 Da) and hydrophobic molecules can pass the BBB *via* simple diffusion while hydrophilic or large molecules in the circulation can pass through active transport and transcytosis.^6^ Many strategies have been developed to deliver drugs and anaesthetics across the BBB.

Physiochemical characteristics of drugs can be manipulated by loading into different nanocarrier systems to target drugs to specific tissues, improve drug bioavailability and biocompatibility, and achieve controlled release.^8,9^ Inorganic and organic nanocarriers were developed for drug delivery which demonstrated great potential to pass the BBB. These nanocarriers were engineered with varying sizes, shapes, and surface modifications to improve their ability to penetrate the BBB and deliver their cargos into the brain.^9–12^ Polymeric nanoparticles were employed for delivery of dopamine to treat Parkinson disease,^8^ asialo-erythropoietin-modified PEGylated liposomes were used for treating cerebral ischemia-reperfusion injury,^13^ and PLGA/polysorbate-80 nanoparticles were delivered to brain tissues *via* the carotid artery.^14^ Dendrimers were used for delivery of drugs for treatment of cerebral palsy and other neuroinflammatory disorders^15^ and doxorubicin was encapsulated in stearic acid-grafted chitosan micelles as a promising brain-targeting delivery system.^16^ Ultrasmall gold nanoparticles (2 nm) surface-modified with fluoresceine were shown to cross the BBB.^17^ Additionally, magnetic nanoparticles coated with heparin were shown to effectively protect neurons from the harmful effect of the accumulation of β amyloid, a hallmark of Alzheimer's disease.^18^ Finally, multiwalled carbon nanotubes were modified with angiopep-2 and used for the transport of anticancer drugs to treat brain glioma.^19^

Propofol, etomidate and ketamine are common anaesthetic agents used in routine surgeries which LOC by acting on gamma-aminobutyric acid and *N*-methyl-d-aspartate receptors.^20–23^ Reduced bioavailability of anaesthetics is due to their short half-life which is caused by the enzymatic degradation in the liver, binding of the drug to plasma albumin, and renal clearance.^24–26^ Additionally, most anaesthetic drugs are lipophilic which makes delivering these drugs *via* intravenous route difficult. Therefore, most of the drugs are either solubilized with lipids or amphiphilic compounds to make nano micelles or nano emulsions.^1,23–37^ LOC time is largely dependent on the dose administered and bioavailability of the drug which are also responsible for the known side effects.

**Fig. 2:**
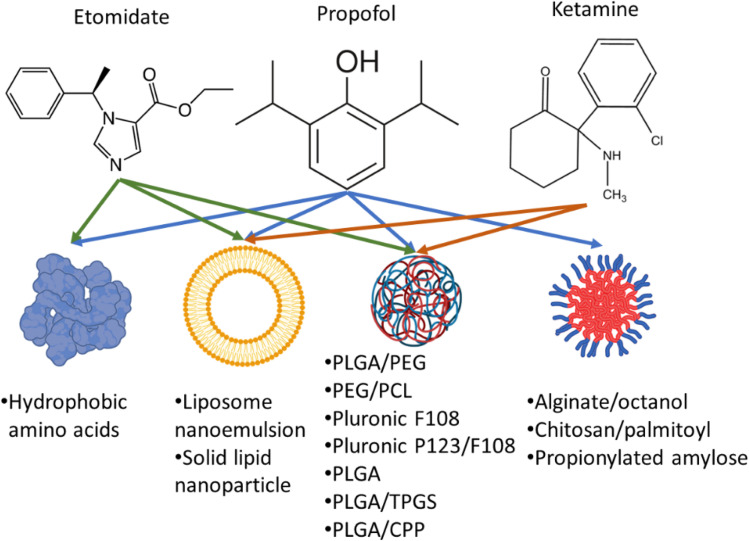
Encapsulation of propofol, ketamine, and etomidate in different nanocarriers. PLGA: Poly(lactic-*co*-glycolic acid), PEG: poly(ethylene glycol), PCL: poly(ε-caprolactone) copolymers, TPGS: d-α-tocopheryl polyethylene glycol succinate, CPP: cell penetrating peptides.

Therefore, nanoformulations to encapsulate anaesthetic drugs were designed to increase bioavailability, and biodistribution in the brain, lower their dose while prolonging the sedation time and decreasing their side effects. Several studies reported the use of different nanocarriers to encapsulate propofol, ketamine, and etomidate (Fig. 2). Tests used to assess the effects of different anaesthetics in animals include loss of righting reflex (LORR) and paw-licking test. LORR is a method to test the onset of loss of consciousness from the time of administration of the anaesthetic drug.^28,30,33,37^ The paw-licking test is used to study pain inflicted on the rat during and after administration. In this review, the platforms used for nanoencapsulation of general anaesthetics will be summarized with a focus on their particle size, encapsulation efficiency, ability to induce LORR and analgesia.

## Propofol

Propofol (Diprivan, Lipuro) is a lipophilic anaesthetic agent which is administered continuously during prolonged surgeries. It is usually composed of 10 mg mL^−1^ of propofol emulsified in soybean oil, glycerol, and egg lecithin. Propofol has been reported to maintain a LORR from 3 to 13 min and a half-life of 0.9 h.^28,30,33,37^ Although propofol induces rapid loss of consciousness, patients recover from the sedation state quickly. However, it causes several side effects (due to its continuous administration) such as lipid overload in the blood and increase susceptibility to microbial infection.^38–40^ The infection occurs extrinsic (handling) due to the microbial agents favouring growth in the presence of lipophilic excipients of the emulsion in Diprivan.^38–40^ Therefore, several studies have explored alternative vehicles to overcome such obstacles for propofol encapsulation (Table 1).

**Table 1:** Nanoencapsulation of propofol^a^

Formulation (particle size; PDI)	Entrapment efficiency (%) (drug concentration)	Animal model & drug injection regimen	Outcome	References
Lipid crystal nanoparticles (soy phosphatidylcholine, glycerol dioleate, polysorbate 80) lipids to polysorbate ratio 80 : 20 (118; 0.22)	10 mg mL^−1^	Male Sprague-Dawley rats, 313 g	LORR duration in the nano formula-treated rats was 448 ± 60 s against propofol-lipuro (10 mg mL^−1^) 377 ± 89 s	33
		Inserted catheter at the jugular vein; a single bolus of formula (total 10 mg kg^−1^ of propofol)	Half-life of nano formula was double of that of lipuro (1.97 h *vs.* 0.90)	
			Clearance from blood was lowered by half in rate treated with nano formula (2799 mL h^−1^) as compared with 10 mg mL^−1^ propofol-lipuro (4326 mL h^−1^)	
(1) ProNano: propofol nano emulsion (sesame oil, capmul MCM, Span 80, Tween 80) ProNano (244 nm; 0.153)	ProNano: 99.50% (10 mg mL^−1^)	Wistar rat male 200–250 g	ProNano and PSNE gave similar LORR duration of 14, and 15 min, respectively	30
(2) PSNE: propofol solid nano-emulsion (capmul, Solutol HS, Tween 80) PSNE (168 nm; 0.32)	PSNE: 100% (250 mg mL^−1^)	LORR regimen: single injection dose 10 mg kg^−1^ at lateral tail vein	ProNano gave the lowest paw-lick time of 6 s among all formulations (22 s for PNS and 14 s for PSNE, and 11 s for Diprivan, *p* < 0.01)	
(3) PNS: propofol nano sponge (Solutol HS, cyclodextrin nanopsonge, glycerol) PNS (510 nm; 0.303)	PNS: 99.86% (10 mg mL^−1^)	Rat paw-lick test regimen: single injection of 100 μL at dose of 10 mg kg^−1^ in the right hind paw	PNS had higher LORR duration of 21 min than Diprivan (13 min; *p* > 0.05)	
Quaternized palmitoyl glycol chitosan (163–200 nm; 0.07–0.13)	Amphiphile	Male MF1 mice, 6 weeks-old, intravenous injection in lateral tail vein (100 μL)	The resulting time of sleep was 0.426 mg min^−1^ when 0.2 mg per mouse was administrated for the formulation against 0.032 mg min^−1^ propofol emulsion	44
	Pluronic F127 (1.78 g L^−1^)	Propofol dose: 0.2, 0.4, 0.5 mg per mouse	0.134 mg min^−1^ when 0.4 mg per mouse was administered for the formulation against 0.023 mg min^−1^ for propofol emulsion	
	Hydroxypropyl-β-cyclodextrin (38.7 g L^−1^)			
Quaternized palmitoyl glycol chitosan attached to G3 dendrimer core (62–182 nm; >0.6)	86% (24 mg mL^−1^)	Male CD-1 mice, 21–27 g	The quaternized palmitoyl glycol chitosan had a significant higher time of LORR of 5.13 min compared to Diprivan of 3.24 min	28
		One bolus injection of 400 μg of 200 μL in lateral tail vein		
Propionylated amylose (55 nm ± 12 nm)	NI	Male rabbits 4.4 kg	Rabbits which received a single bolus dose of propionylated amylose showed rapid onset of reaching alertness state but slow onset in LORR in contrast to free Diprivan (fast in LORR and slow onset of reaching alertness state)	25
		Venous cannulation at marginal ear vein 4.5 mg kg^−1^ single bolus	However, maintaining LORR was shorter in propionylated amylose group	
		Continuous bolus 30 mg kg^−1^ h^−1^ then decreased to 9 mg kg^−1^ h^−1^ then decreased to 4.5 mg kg^−1^ h^−1^		
		Male nude mice, 20 g	As showed by imaging studies, propionylated amylose reached the brain within 3 min then gradually decreased till 30 min post injection. It reached other organs and was especially concentrated in the liver	
		Injected 0.1 mL once in lateral tail vein (50 μL mL^−1^)		
Alginate/octanol 1% w/v (180 nm; 0.21)	99.4% (5 mg mL^−1^)	Male Sprague Dawley rats, single dose of 10 mg kg^−1^ of drug	Reaching LORR took 35 s for both the nano formulation and Diprivan	32
		Intravenous injection in the caudal vein	Rats recovered from LORR after 7.18 min for nanoformulation and 7.25 min for Diprivan	
PEG-PCL copolymer (320 nm)	11.8% (177 μg mL^−1^)	Adult male Fischer 344 rats 150–200 g	Half-life: first phase 8.8 min, second phase 270 min	24
The nano formulas were doped with infrared fluorescent dye		Injection *via* lateral tail vein using tail vein catheter total volume 1 mL	Biodistribution: liver, spleen, and lung	
PEG (2 kDa): PLGA (5 kDa) 150 mg of polymer to 15 mg of propofol (397.3 nm; 0.068)	(1.5 mg mL^−1^)	Male Long-Evans rats 180–200 g	Whole blood clearance showed half-life of propofol drug is 12.4 min and 91.4 min in plasma	26
		1 bolus 1 mg kg^−1^ of drug intravenously infused using catheter 24gx3/4′′ inside rat lateral tail vein total volume was 0.4–0.5 mL	Biodistribution: most of the drug was in liver, heart, and lungs. The brain, however, showed AU of <0.2 and the least tissue to have propofol	
		Blood samples were collected from submandibular vein at different time points (2, 10, 20, 40 min, 2 h, 4 h)		
Self-assembled hydrophobic amino acids of GQQQQQY sequence (293 nm)	96.59% (10 mg mL^−1^)	Male Sprague Dawley rats, 8 weeks-old, 230–340 g. Weaning 2 weeks-old rats, 101–114 g	LORR onset achieved in nanoformulation was 0.2 min against Diprivan 0.14 min	37
		Nanoformulation and free propofol were administered *via* venepuncture in the tail lateral caudal vein	LORR duration was 8.33 min against Diprivan was 9.16 min	
		Single bolus dose at a rate of 0.1 mL s^−1^ for a total of 0.6 mL	Sedation time with the nanoformulation was 13.6 min while with Diprivan was 12.6 min	
			Paw lifting and paw licking were reduced in nanoformulation treated group (33.3% and 0%) as compared to animals treated with free Diprivan (100% and 66.7%)	

a NI: not investigated; G: glycine; Q: glutamine; Y: tyrosine.

### Encapsulation of propofol in lipid nanoparticles

Lipid nanoparticles can bypass the BBB and release their anaesthetic cargo in a slow manner which could prolong the LORR effect. They are prepared of a lipid moiety and a stabilizer of varying molecular sizes such as polysorbate 80 or Tween 80. The homogenization or sonication of the mixture in water creates an emulsion ideal for delivering hydrophobic drugs such as propofol.^33,35,41–43^ Johnson *et al.*, used soy phosphatidylcholine, glycerol dioleate, and polysorbate to create a lipid crystal nano emulsion with a particle size around 118 nm.^33^ The propofol concentration in this nano formula was 10 mg mL^−1^ (which is similar to propofol concentration in commercial Lipuro) which exhibited a LORR duration of 7.5 min compared to 6.3 min for Lipuro. However, propofol in lipid nanocrystal had a longer half-life time of 0.9 h as compared to 2.9 h for Lipuro in Sprague Dawley rats.^34^ This was due to the clearance of the propofol in the lipid nanocrystal was almost half (2799 mL h^−1^) that of Lipuro (4326 mL h^−1^). The study, however, didn't explore the pain threshold of their nano formulations or the biodistribution of propofol in different tissues such as the brain, liver, lungs, and kidneys.

Darandale *et al.* developed two distinct lipid-based nano formulations: propofol nano emulsion (ProNano) and propofol solid nano emulsion (PSNE).^30^ A third lipid-free nanoformulation was also prepared and named propofol nano sponge (PNS). ProNano demonstrated a lower free propofol concentration (0.13%) which reduced injection pain as compared to Diprivan. ProNano and PSNE showed LORR duration of 14 min and 15 min, respectively, as compared to LORR of 13 min for Diprivan. Additionally, the lipid content of the medium-chain glyceride-based nano emulsion (ProNano) was low, potentially lowering the risk of hyperlipidemia (a Diprivan side effect). On the other hand, PNS demonstrated a longer LORR duration of 21 min which may be related to the sustained release feature of PNS.^30^ On the other hand, paw licking was decreased in animals administered propofol in lipid nanoparticles as compared to those which received Diprivan which indicates low pain induction upon injection. Propofol nanoencapsulation may reduce susceptibility to infection related to propofol immunomodulatory effects.^30^ More studies, however, are needed to evaluate the systemic biodistribution of nano encapsulated propofol especially in the CNS and assess its potential in reducing susceptibility to microbial infection.

### Palmitoyl quaternary ammonium glycol chitosan nanoparticles

Quaternary ammonium chitosan (QCS) is prepared by replacing amine group of chitosan with quaternary structures forming a potent positive charge within the chitosan molecule. QCS is water soluble regardless of the pH. However, to load propofol, a hydrophobic moiety would be needed. For this purpose, two studies employed palmitoyl oil or dendrimer core and showed similar entrapment efficiency and small nanoparticle size.^28,44^ However, the dendrimer core resulted in variation in nanoparticle size of QCS, PDI >0.6 (Table 1). Despite their poor homogeneity, the dendrimer core QCS exhibited a lower LORR in mice upon administration when compared to Diprivan.^28,44^

### Alginate grafted nanoparticles

Alginate nanoparticles are used for drug delivery for several reasons: biodegradability, enhanced bioavailability of the drug, and ability to encapsulate hydrophobic drugs with great stability. Propofol was encapsulated in alginate nanoparticles grafted with octanol.^32^ The encapsulation efficiency was 99% with a nanoparticle size of 80 nm as evident by TEM. The nanoparticles developed also achieved sustained release of 20% in 2 h and 72% in 48 h with an onset of LORR of 35 s. This study demonstrated a promising nanoparticle delivery system but lacked the safety studies of haemolysis for octanol-alginate, pain response, and its biodistribution. It is of note, however, that sodium alginate was reported to have a generally low haemolytic activity.^45^

### Propionylated amylose nanoparticles

**Fig. 3:**
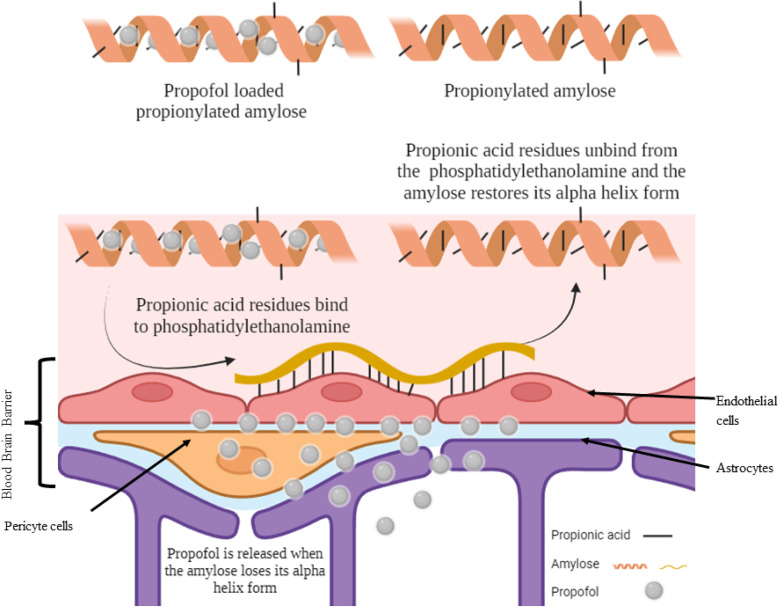
Targeting propofol to the blood–brain barrier using propionylated amylose. Reproduced from Gao *et al.* with permission from [Elsevier], Copyright [2017].^25^

Propionic acid is the smallest fatty acid which can attach to phosphatidylethanolamine found in the endothelial cells of the BBB.^25^ A hydrophobic system was developed using propionylated amylose (PPA) to post-load propofol and actively target propofol to the BBB (Fig. 3). The particle size of the PPA was 55 nm. The propofol in PPA reached the brain in 3 min and was persistent for 30 min, then the PPA started to distribute through the liver, lungs and finally to the kidneys. The PPA developed reduced the dose needed for propofol to induce LORR and even induced a faster LORR onset time of up to 51 s. However, the LORR was kept for only 5 min as the nanoparticle exhibited a burst release of propofol upon reaching the BBB.^10^

### Synthetic block copolymers

Synthetic block copolymers are amphiphilic polymers that can encapsulate hydrophobic drugs. Pluronic, poloxamer, PEG-PLGA, and PEG-PCL are examples of amphiphilic polymers that can be suspended in phosphate-buffered saline and encapsulate hydrophobic drugs.^24,26,27^ PEG-PCL and PEG-PLGA polymers were used to encapsulate propofol in normal saline and resulted in nanoparticle size range of 320–398 nm as measured by dynamic light scattering (DLS) with a PDI of 0.056. It is of note that DLS readings for nanoparticle size are always higher than those measured by TEM due to the hydration shell phenomenon.^46^ Most of the nanoparticles administered resided in the liver, lungs, and spleen while the brain had the least amount of propofol.^24,26^ The nanoparticles developed showed a sustained release and a half-life of 91–270 min. A comparison of biodistribution of the nano encapsulated Diprivan *versus* free Diprivan could have led to a better understanding of whether the developed nanoparticles were superior in passive targeting than free Diprivan. Additionally, animal studies to investigate the effects of propofol-polymer nanoformulation on LORR and paw-licking values are also needed to assess the efficacy of the proposed nanosystem.

### Hydrophobic peptides

Synthetic peptides containing a hydrophobic motif self-assemble forming nanoparticles.^47^ Specifically, peptides containing GQY amino acid motif were shown to efficiently encapsulate hydrophobic drugs including general anaesthetics such as propofol and etomidate.^37^ The reported entrapment efficiencies were 96.5% and 78.8% for propofol-GQY and ET-GQY, respectively. Nanoparticle size was less than 100 nm and showed promising homogeneity and stability and caused less pain upon injection. Moreover, haemolysis and cytotoxicity tests indicated safety of GQY loaded with propofol and ET for intravenous administration. GQY loaded with propofol and ET induced similar LORR timings and were deemed as efficient as free Diprivan and Forry. However, the pH reported in this study was highly acidic at 2.92, 4.23 and 6.01 for propofol, ET, and ET26 (an analogue of etomidate), respectively, which could be dangerous if administered intravenously.^37^ Moreover, the GQY system showed a few side effects in rats such as myoclonus. Therefore, further investigation is needed to ensure the safety and reduce the acidity of GQY formulations loaded with propofol or ET.

## Etomidate

Etomidate is a lipophilic anaesthetic drug which targets gamma-aminobutyric acid-A receptors. In addition to its rapid onset, etomidate minimally affects breathing and has hemodynamic stability making it especially suitable for cardiovascular and critically ill patients. Etomidate is 1.8-fold more potent than propofol.^37,41,48,49^ However, etomidate lipid emulsions have several drawbacks. It causes pain upon injection due to its poor water solubility. It inhibits 11β-hydroxylase leading to the suppression of the adrenocortical axis. It also causes postoperative nausea and vomiting.^48,49^ Etomidate is rapidly metabolized by hepatic esterase and its metabolites are excreted in urine. Etomidate nanoformulation and analogues have been developed aiming to retain its stability for cardiorespiratory profile whilst overcoming the side effects.^49^

Etomidate is marketed as Etomidate-Lipuro® (B-Braun, Germany), and ForryTM (Nhwa, China) which were used in studies presented in this review.^41,49^ The marketed etomidates are oil-in-water emulsions consisting of soybean phospholipids and egg lecithin, and propylene glycol as a cosolvent forming etomidate fat emulsions of 168 nm particles with low PDI.^41^ Lipurom and Forry have a LORR onset of 8 to 12 s and a duration of 8–11 min with a half-life of 2.9–5.5 h.^37,48,49^ Commercial fat emulsions have mean diameters range of 200–400 nm.^42^ The studies investigated etomidate encapsulation focused on lipid nanoparticle formulations to reduce its side effects and improve stability of cardiorespiratory profiles of patients receiving the drug. The most important findings from these studies are summarised in Table 2.

**Table 2:** Selected etomidate nanoencapsulation studies^a^

Formulation (particle size; PDI)	Entrapment efficiency (%) (drug concentration)	Animal model and injection regimen	Outcomes	References
Soybean oil and medium chain triglyceride in ratio 1 : 1 of 10.07% all dissolved in organic phase. Egg lecithin of 1.56%, Pluronic F68 0.34% sodium oleate 0.05% and glycerol 2.25%	97.65% ± 0.16% (2 mg mL^−1^)	Male Sprague Dawley (SD) rats (200 g ± 20 g)	Vascular irritation tests showed light vasodilation inside tissue compared to the marketed etomidate showing vasodilation, inflammatory cellular infiltration, and edema	41
		One bolus of intravenous *via* tail vein administered 5 mg kg^−1^ of etomidate	Etomidate of lipid nano emulsion and marketed etomidate were cleared from the system 2 min after administration	
		Japanese white rabbits (2.5 kg ± 0.5 kg)	Marketed etomidate had a higher penetration of the brain than the lipid nano emulsion (1.52 μg mL^−1^*vs.* 2.99 μg mL^−1^)	
		Administered 1 mg kg^−1^ delivered intravenous *via* right ear margin vein	The lipid nano emulsion was found in the liver, lung and heart (4.48, 4.90, and 3.11 μg mL^−1^, respectively)	
60 mg of Pluronic P123 and 60 mg of Pluronic F108 in 5 mL of chloroform, evaporated under rotary evaporator. (33.8 nm; 0.231)	86.04% (2 mg mL^−1^)	Male SD rats (250–300 g)	The onset of the LORR reduced by increasing the drug dosage from 11.7 s for 2 mg kg^−1^ of etomidate to 8.8 s for 3 mg kg^−1^ of etomidate	51
		One bolus intravenous injection of 2, 2.5, or 3 mg kg^−1^ of etomidate	The duration of LORR is increased due to increase of dosage 608 s for 2 mg kg^−1^ of etomidate to 791 s for 3 mg kg^−1^ of etomidate	
			The nano emulsion was unstable and doubled in size after 50 days and increased to >200 nm after 180 days	
120 mg Pluronic F108, 0.5 mg soybean oil, and 10 mg of etomidate (thin film hydration method). (109 nm; 0.296)	86.69% (2 mg mL^−1^)	Adult male Sprague Dawley rats (200–300 g)	Etomidate in poloxamer micelles resulted in LORR duration of 8.5 min as compared to etomidate in fat emulsion of 9.4 min	48
		One bolus intravenous injection 2 mg kg^−1^ (0.2–0.3 mL)	The onset of LORR were 10 s after IV injection for both preparations	
60 mg of Pluronic P123 and 60 mg of Pluronic F108 in 3 mL of chloroform evaporated under rotary evaporator. (40.5 nm; 0.25)	86.46% (2 mg mL^−1^)	Male adult SD rats (220–300 g)	LORR onset of etomidate nano emulsion was 11.3 s *vs.* etomidate fat emulsion of 9.75 s	52
		One bolus intravenous injection dose of 2 mg kg^−1^ of etomidate at a rate of 1 mL min^−1^	The duration of LORR of etomidate nano emulsion was 609 s as compared to 581 s of commercial etomidate	
			Toxicity of etomidate nano emulsion *in vivo* has an 0.53 OR of death *vs.* fat emulsion at 20 mg kg^−1^ (*X*^2^ = 0.55)	
			No precipitation was observed after 15 days for this formula	
GQQQQQY (hydrophobic amino acids) (306 nm)	78.80%	Male SD rats 8 weeks old, 230–340 g. Weaning 2 weeks old rats 101–114 g	ET-GQY and the commercial etomidate showed almost similar onset of action, duration of LORR, and sedation time	37
		Nanoformulation and commercial propofol were administered *via* venepuncture in the tail lateral caudal vein	The pH of ET-GQY was 4.2 as compared to 6.0 for Forry (commercial etomidate) and 6.01 for ET26 (etomidate analogue). This makes ET-GQY unsuitable for IV injection	
		Single bolus dose (rate of 0.1 mL s^−1^ for a total of 0.6 mL)		

a ET: etomidate, G: glycine, LORR: loss of righting reflex, Q: glutamine, SD: Sprague Dawley, Y: tyrosine.

### Solid lipid nanoparticles (SLN)

The first trials for etomidate nanocarriers were in the form of etomidate-loaded solid lipid nanoparticles with convenient loading capacity of 10% with a burst drug release of 100% release in 1 min.^43^ The particle mean diameter determined by DLS was 140–180 nm with a pH between 5 and 7 which gave the highest physical stability. Cholesteryl myristate (CM) was used to form nanoparticles with a smaller size of 100 nm (PDI range of 0.14–0.16) with greater stability.^50^ Finally, intravenous lipid emulsions were developed and exhibited a similar pharmacokinetic profile to commercial etomidate as it showed low haemolysis but with low vascular irritation.^41^

### Poloxamer micelles

Poloxamer micelles were prepared from Pluronic F108 and Pluronic P123 micelles using thin-film hydration and loaded with 2 mg mL^−1^ etomidate.^34,35^ Etomidate release from the poloxamer micelles was slower (68% in 6 h) than from the commercial etomidate fat emulsion (86% in 6 h).^34^ The average particle size measured using DLS, of poloxamer micelles prepared from Pluronic F108 and loaded with etomidate was 109 nm which was reduced to 40.5 nm, with PDI ranging from 0.250–0.296, upon adding Pluronic P123.^34,35^ For etimodate loaded in poloxamer micelles, the onset times of LORR in rats were 11 s, 10 s, and 8.8 s for 2.0, 2.5, and 3 mg kg^−1^ etomidate.^34^ Moreover, the duration of LORR increased in a dose-dependent manner (10, 11.8, and 13.1 min respectively). Wu *et al.* used soybean oil to modulate the hydrophobicity of Pluronic F108 micelles to achieve a stable encapsulation of etomidate. However, this formulation (containing 2 mg etomidate per kg) gave a shorter LORR duration of 8.5 min as compared to 9.4 min for commercial etomidate.^31^

## Ketamine

Ketamine (brand names Ketalar or Ketamav) is another general anaesthetic drug is used during routine surgery and also as an analgesic for pain relief. The analgesic effect is also important as ketamine is known to regulate morphine and its analogues tolerances in the central nervous system. However, it is a lipophilic drug with very limited water solubility (1 mg mL^−1^ of 5% dextrose or 0.9% saline) and hence low bioavailability in circulation with a half-life of 0.66–0.8 h.^53,54^ Studies that investigated ketamine focused more on pain relief rather than loss of consciousness and used intrathecal route of administration to bypass the blood–brain barrier and avoid complications that may occur upon intravenous administration of nanoparticles. Details of studies which investigated nanoencapsulation of ketamine are presented in Table 3.

**Table 3:** Studies on nanoencapsulation of ketamine

Formulation (particle size; PDI)	Entrapment efficiency (%)	Animal model	Outcomes	References
PEG 5 kDa/PLGA 55 kDa (98.8 nm; 0.18 ± 0.01)	64 ± 2.5% for PEG/PLGA	Male C57BL/6J mice (aged between 10–12 weeks)	- PEG/PLGA: Shellac was cleared from blood after 5 days	53
PEG 5 kDa/PLGA 55 kDa: Shellac (107.4 nm; 0.18 ± 0.01)	71.8 ± 1.2% for PEG/PLGA: Shellac	Single intravenous bolus 1 mg kg^−1^ of ketamine *via* lateral tail vein	- Most of the nanoparticles were distributed in the liver, brain and kidneys	
			- Half-lives of PEG/PLGA, PEG/PLGA: Shellac, and ketamine suspension *in vivo* were 103, 79.7, and 0.6 h, respectively	
PLGA TPGS (tocopherol polyethylene glycol succinate) (109 nm; 0.296)	86.69%	Adult male local rabbits	-The nanoparticle administered a late onset of LORR than regular drug cocktail of ketamine and xylazine	58
		Intramuscular single injection 30 mg kg^−1^ of ketamine nanoparticle with 10 mg kg^−1^ of xylazine	- The recovery from LORR was prolonged in nanoparticle administered than regular drug suspension	
		Another group with 15 mg kg^−1^ and 5 mg kg^−1^ of xylazine	- No significant difference in muscle relaxant against normal anaesthetic suspension	
1,2-Distearoyl glycero-3-phosphocholine: cholesterol (9 : 1)	65.6%	C57BL/6J male mice (10–12 weeks old)	Half-life time of ketamine increased from 0.88 h to 24 h	54
Liposomes were prepared by thin-film method		Dose: 5% glucose containing 1 mg kg^−1^ ketamine solution or ketamine liposome injected intravenously *via* lateral tail vein	The ketamine nanoparticle distributed evenly (1800 ng g^−1^ of tissue) in liver, brain, and kidney after 5 days	
Post loading of 300 mg ketamine in 10 mL of liposome. (738 nm; 0.44)			- Ketamine nanoparticles clearance was 0.49 μg mL^−1^ h^−1^*vs.* free ketamine at 1.48 μg mL^−1^ h^−1^	

### PLGA

PLGA has been thoroughly studied for ketamine's encapsulation, as it is a biodegradable polymer suitale for drug delivery. Ketamine can be dissolved readily in organic solvents, therefore easy to prepare through solvent-antisolvent system, or through salting out preparation.^53,55–61^ Moreover, it can be easily functionalized *via* apolipoprotein E (Apo-E) and vitamin E (tocopherol) for active drug targeting.^56,60^ Xu *et al.* used PLGA to encapsulate porous silica containing ketamine with larger particle size (50–60 μm) for direct intrathecal administration of ketamine towards the CNS.^59^ However, the study lacked *in vivo* assessments to demonstrate the safety of the porous silica on the caudal nerve and the efficiency of analgesia.

Targeting moieties and particle size of PLGA nanoparticles are important factors to facilitate passing the BBB. The reported PLGA-ketamine nanoparticle sizes ranged between 60–500 nm. Han *et al.* and Bader *et al.* used Shellac (a water soluble resin used in food industry) and vitamin E to improve homing of the nanoparticle towards the brain.^53,60^ As Han *et al.* demonstrated that the PEG-PLGA nanoparticles were well distributed inside the brain, liver and lungs. Similar results were reported for PEG-PLGA coated with Shellac.^53^ Moreover, the study investigated the haematological profiles of the mice and demonstrated that they demonstrated good safety profile against polymeric materials.

### Liposomes

Liposomes are commonly used for delivery of several drugs. They are made up of synthetic or natural phospholipids. These phospholipids self-assemble into small vesicles where the hydrophobic tails of the phospholipids face the inner lamellar structure, and the hydrophilic heads face the core of the vesicle and the outer part of the vesicle. Liposomes are either unilamellar (consisting of a single bilayer of phospholipids) or multilamellar. Hydrophilic drugs can be encapsulated in the core of the vesicles while hydrophobic cargo is loaded within the lamellar structure.^62^ Moreover, liposomes can be engineered to target certain cell types by decorating their surfaces with antibodies, carbohydrates, proteins, small peptides, or small molecules.^62^ Functionalization with immunoglobulin G, transferrin, or leptin were shown to facilitate entry of liposomes across the BBB *via* receptor-mediated transport.^62–65^ One study reported preparation of multilamellar vesical liposome (MVL) and post loaded the ketamine achieving around 65.6% EE. A complete release of ketamine was achieved under 8 h, but the half-life of ketamine *in vivo* was 23.97 h in comparison to the commercial ketamine of 0.6 (ref. 37) and 0.88 h.^54,55^ The liposomes were found mostly in liver, brain, and kidneys and the ketamine concentration in serum was within detectable limits even after 5 days.^54^ However, analgesic effectiveness, onset and duration of LORR, and haemolysis data were not reported.

## Conclusions and future perspectives

Anaesthetic drugs are used for routine surgery or for prolonged analgesic affects for terminal patients where patients develop side effects and tolerance to the administered drugs. Therefore, nanoformulation of anaesthetics was investigated to enhance safety, achieve sustained release, increase half-life time, and analgesic effectiveness upon bolus administration. However, not all nano systems work for different anaesthetics such as propofol, etomidate and ketamine. For example, loading propofol in solid lipid nanoparticles, without a surfactant, results in its crystallization. For etomidate, nano formulations (solid lipid nanoparticles and Poloxamer micelles) studied didn't improve the LORR onset or duration in rats. Also, they resulted in a burst release response rather than sustained release. In the case of ketamine, the nano formulations studied (PLGA and liposomes) showed effectiveness for 3–6 h. Therefore, further studies are needed for different formulations that prolong sustained release, enhance anaesthetic capabilities, and reduce the drug dose to reduce the side effects.

On the other hand, there were interesting formulations used that could have been further studied in terms of preclinical and clinical trial setups to ensure safety of the nano formulations; such as hydrophobic peptide of GQQQQQY, and propionylated amylose. In the case of the propionylated amylose it was developed for targeting the type of phospholipids that exist only in the BBB and is considered the most plausible method for drug targeting and dose reduction. Moreover, burst release ensures fast anaesthetic response and prolongs the anaesthetic state for 30 min. More studies can be extended towards other anaesthetic drugs to test their efficiency. In the case of the hydrophobic amino acids that were used for etomidate and propofol, it was designed to reduce the use of lipophilic excipients and consequently reduce infection and induction of pain upon injection. The hydrophobic peptide also reduced the onset of LORR more than that induced by commercial products. Investigations could be further extended towards preclinical studies to ensure safety of the nanoformulations.

Loading propofol in PEG-PLGA nanoparticles resulted in propofol biodistribution mostly in the liver, lungs, and kidneys.^26^ On the other hand, loading ketamine in PEG-PLGA nanoparticles showed biodistribution of the nanoparticles mainly in brain, liver, and kidneys.^53^ Therefore, more investigations are warranted to clarify whether the cargo (propofol, ketamine, or etomidate) could result in variable biodistribution of the PEG_5KDa_-PLGA_55kDa_ nanoparticles.

Further studies are warranted to improve the performance and reduce side effects of general anaesthetic drugs as this would increase surgery success and pain relief while decreasing morbidities. Nanoencapsulation of anaesthetics has the potential to achieve such goals. A focus on developing nanocarriers capable of encapsulating anaesthetic drugs with high efficiency and crossing the BBB should be attempted. Additionally, designing nanoformulations which could be administered intranasally could serve as a better alternative to intrathecal administration of anaesthetics (although a well-established practice in surgery but causes severe complications which could outweigh its benefit). Lastly, preclinical trials, using larger animals, are needed to assess effectiveness of nano formulated anaesthetics, morbidities, side effects, and pain inflicted upon injection.

It is of note that nanoparticle-based systems were also developed for delivery of local anaesthetics. Porous silica nanoparticles were utilized to achieve controlled release of ropivacaine. Glycosylated chitosan encapsulated mesoporous silica nanoparticles were designed to induce prolonged analgesia in response to ultrasound irradiation.^66^ Additionally, hollow mesoporous organosilica nanoparticles, containing organic groups across the inorganic silica scaffold, were developed for controlled and sustained release of loaded ropivacaine for sustained local anaesthesia. The mesoporous organosilica nanoparticles can be repeatedly triggered to release the anaesthetic cargo in response to ultrasound irradiation or low pH, resulting in long-lasting analgesic effect.^67^ Finally, tetrodotoxin, a strong local anaesthetic, was loaded into hollow silica nanoparticles to extend its nerve blockade and lower its toxic side effects.^68^

## Abbreviations

BBB: Blood brain barrier
Et: Etomidate
I.T.: Intrathecal
IV: Intravenous
LOC: Loss of consciousness
LORR: Loss of righting reflex
QCS: Quaternary ammonium chitosan
NP: Nanoparticles

## Author contributions

BA and SA wrote the first draft. H. M. E. A. guided and supervised the first draft and revised the final draft.

## Conflicts of interest

There are no conflicts to declare.

## Supplementary Material
